# AbImmPred: An immunogenicity prediction method for therapeutic antibodies using AntiBERTy-based sequence features

**DOI:** 10.1371/journal.pone.0296737

**Published:** 2024-02-23

**Authors:** Hong Wang, Xiaohu Hao, Yuzhuo He, Long Fan

**Affiliations:** 1 Production and R&D Center I of Life Science Services, GenScript Biotech Corporation, Nanjing, China; 2 Production and R&D Center I of Life Science Services, GenScript (Shanghai) Biotech Co., Ltd., Shanghai, China; Albert Einstein College of Medicine, UNITED STATES

## Abstract

Due to the unnecessary immune responses induced by therapeutic antibodies in clinical applications, immunogenicity is an important factor to be considered in the development of antibody therapeutics. To a certain extent, there is a lag in using wet-lab experiments to test the immunogenicity in the development process of antibody therapeutics. Developing a computational method to predict the immunogenicity at once the antibody sequence is designed, is of great significance for the screening in the early stage and reducing the risk of antibody therapeutics development. In this study, a computational immunogenicity prediction method was proposed on the basis of AntiBERTy-based features of amino sequences in the antibody variable region. The AntiBERTy-based sequence features were first calculated using the AntiBERTy pre-trained model. Principal component analysis (PCA) was then applied to reduce the extracted feature to two dimensions to obtain the final features. AutoGluon was then used to train multiple machine learning models and the best one, the weighted ensemble model, was obtained through 5-fold cross-validation on the collected data. The data contains 199 commercial therapeutic antibodies, of which 177 samples were used for model training and 5-fold cross-validation, and the remaining 22 samples were used as an independent test dataset to evaluate the performance of the constructed model and compare it with other prediction methods. Test results show that the proposed method outperforms the comparison method with 0.7273 accuracy on the independent test dataset, which is 9.09% higher than the comparison method. The corresponding web server is available through the official website of GenScript Co., Ltd., https://www.genscript.com/tools/antibody-immunogenicity.

## Introduction

With the continuous development of the pharmaceutical industry, the development of therapeutic proteins is growing rapidly. Monoclonal antibodies account for nearly half of the growing number of therapeutic proteins approved by the U.S. Food and Drug Administration (FDA) [1]. Therapeutic antibodies can be used for targeted treatment of chronic diseases, autoimmune diseases, cancer, etc [2, 3]. Immunogenicity of therapeutic antibodies refers to the presence of anti-drug antibodies (ADAs) detected in the circulatory system of humans or antibodies that bind to the antibody drug that has been injected. The immune mechanism of B cell activation leading to ADAs secretion includes T cell-independent (Ti) and T cell-dependent (Td) conditions. Td activation of B cells is thought to lead to a stronger immune response, antibody type switching, and the production of memory B cells [4]. Because the Td reaction requires T cells to recognize linear antigenic peptides (T cell epitopes) contained in antibody drugs, binding of peptide epitopes processed by antigen-presenting cells (APCs) to human leukocyte antigen (HLAs) major histocompatibility complex (MHC) Class I or II molecules may occur. Activated helper T cells recognize epitope-MHC I or II complexes to stimulate B cells to produce ADAs [4, 5]. The generation of ADAs is gradually considered to be one of the reasons for the development failure of some antibody drugs, which may cause a variety of problems, including changing the pharmacokinetics of drugs, reducing drug activity, and even causing life-threatening complications, affecting drug safety and efficacy [6–10]. Therefore, evaluation of immunogenicity is an important issue to be considered in the process of drug development for therapeutic antibodies [11]. Researchers have tried to use the humanization of antibodies as an important strategy to reduce ADAs production. However, the correlation between the degree of humanization of antibodies and the presence of ADAs is relatively weak [12]. Traditional antibody immunogenicity detection methods rely on immunological and biochemical experiments, which are costly and time-consuming [13]. In-silico and immunoinformatic analysis-based methods are able to avoid these shortcomings to a large extent [14].

On the basis of the immune response mechanism, most of the existing computational methods predict MHC binding, T cell epitopes and B cell epitopes for inferring the immunogenicity [15]. Given the critical role of CD4+ T cell epitopes in immune response, Oyarzun et al. developed Predivac [16]. Predivac uses the constructed PredivacDB database to calculate the correlation between specific determinative residues (SDRs) in HLA query proteins and known HLA protein-associated SDRs, thereby predicting the high binding affinity of HLA II peptides and CD4+ T cell epitopes. Bhasin et al. developed a method for predicting MHC I-restricted T cell epitopes from antigen sequences, CTLpred [17], based on quantitative matrix (QM), support vector machine (SVM) and artificial neural network (ANN). Sweredoski et al. proposed PEPITO [18] and COBEpro [19] to predict discontinuous and linear B cell epitopes, respectively. PEPITO [18] calculates epitope scores based on the linear combination of amino acid propensity score and multi-distance hemispherical exposure values. COBEpro [19] uses SVM to predict epitope propensity of short peptide fragments and amino acid residues in antigen sequences based on sequence similarity, secondary structure, and solvent accessibility characteristics. Liang et al. used support vector regression (SVR) to construct linear and discontinuous B-cell epitope prediction models, EPSVR [20], by calculating six features, such as residue epitope propensity, side chain energy score and conservatism, and took the area under the receiver operating characteristic curve (*AUC* score) as an evaluation index to prove its good prediction performance. However, nearly all existing TCR and BCR epitope prediction tools are not directly used to predict the clinical immune response of antibody drugs after injection into the body; only one TCR epitope prediction method is found for this [21], but not benchmarked.

There are few tools available to predict the clinical immunogenicity of antibody drugs at present. The only one computational method, PITHA [22], was proposed by Liang et al. in 2022. PITHA was constructed based on a SVM classifier and Leave-One-Out Cross-Validation (LOOCV) method to distinguish high/low antibody immunogenicity, which extracted the characteristics of B cell epitopes, including the cavity volume at the CDR region and hydrophobicity of the CDR-H3 loop and the glycine number at the CDR-H2 loop. PITHA compared the results of models trained using different feature combinations. Verification results of LOOCV show that when using the cavity volume at the CDR region and hydrophobicity of the CDR-H3 loop features, an *accuracy* of 0.83 can be achieved on the training dataset with crystal structures. Independent testing showed that PITHA could get 0.65 in terms of *accuracy* on the test dataset with modelled structures. Although PITHA has been developed to predict high/low clinical immunogenicity of therapeutic antibodies, there still exists several problems that affect the application of it: **(1)** the traditional feature engineering relies on manual design and calculation, which is not only complicated and time-consuming, but also has a great impact on the performance of classification algorithms; **(2)** 3D structure of antibodies is necessary for making such prediction. To get the 3D structure, one can choose the expensive and time-consuming traditional experimental methods or the less accurate computational techniques, while both of these methods have their own defects; **(3)** the data set used in the existing methods is considerably small.

In order to overcome the above-mentioned limitations, **(1)** Natural Language Processing (NLP) has been introduced to the biological field by treating biological sequences as sentences: some Pre-trained Protein Language Models (PPLMs) such as ESM [23], ProtBERT [24], ProtT5-XL-UniRef50 [24], ProtNLM [25], Unirep [26], and Pre-trained Antibody Language Models (PALMs) such as AntiBERTy [27], AntiBERTa [28], and EATLM [29] have been trained [29, 30]. The use of BERT and other transformer-based language models for protein sequence representation has been proven to be effective in epitope prediction as well as in predicting binding affinity between MHCs and peptides [31, 32]. The improvement of the immunogenicity-related prediction task using BERT and other pre-trained language models indicates that it is possible to construct predictive models of immunogenicity for therapeutic antibodies with the use of pre-trained PLMs. Different from general protein language models, AntiBERTy is obtained by training on a large number of antibody sequences, which can capture more information and features in the antibody sequence than some hand-designed features; **(2)** prediction without 3D structures is also available, because the variable region of an antibody is the most critical component of the antibody molecule that is responsible for binding with antigens. The extracted features from the variable region of antibody with the use of AntiBERTy have great potential to predict the immunogenicity of antibodies more accurately; **(3)** there are now more than 100 commercial antibody therapeutics and their immune response data in the population. With the increase of the data, it is increasingly possible to predict the clinical immune response.

**Fig 1:**
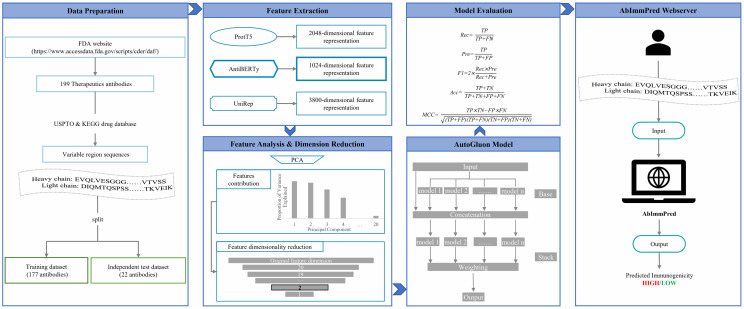
The workflow of AbImmPred. The workflow consists of five parts: Data Preparation; Feature Extraction; Feature Analysis and Dimensionality Reduction; AutoGluon Model; Model Evaluation; and AbImmPred Webserver. The obtained dataset was first divided into training and test parts. Three pre-trained models, ProtT5, AntiBERTy, and UniRep were used to extract features then. PCA was next used to compress the high-dimensional features extracted by the three pre-trained models to a reasonable level, and AutoGluon was hereafter used to build models and evaluate their performance. Finally, an online server was built and provided.

In this study, a computational immunogenicity prediction method, AbImmPred (**A**nti**b**ody **Imm**unogenicity **Pred**ictor), was proposed to predict the high/low immunogenicity of therapeutic antibodies from only the variable region amino acid sequences. The main process of the work is shown in Fig 1. For feature extraction, based on the amino acid sequence of the variable region of the antibody, the encoder in the AntiBERTy pre-trained model was used, and the output of embedding was regarded as the sequence features. The extracted features were reduced to two dimensions by PCA to obtain the final features. AutoGluon was used to train a series of basic machine learning models, and based on that a weighted ensemble model was trained. The optimal model was determined through 5-fold cross-validation [33]. The model constructed in this study was based on 199 commercial therapeutic antibodies, of which 177 samples were used for model 5-fold cross-validation and final model training, and the remaining 22 samples were used as an independent test dataset to evaluate the performance of the constructed model and compare with other prediction method. The results on the independent test dataset show that AbImmPred has an *accuracy* of 0.7273, which is significantly higher than the existing method (0.6364). In addition, our model was evaluated in four other indicators: *recall* (0.9375), *precision* (0.7500), *F1-score* (0.8333), and *MCC* (0.1614), in which *recall* and *F1-score* were higher than the existing methods. Due to the good performance of AbImmPred in predicting the immunogenicity of therapeutic antibodies, the proposed method could potentially speed up the development process of antibody therapeutics.

## Results

### Feature analysis and dimension reduction results

Feature analysis and dimension reduction results are first presented. UniRep, ProtT5, and AntiBERTy language models were used for extracting the features which were then analysed and compressed by PCA that was constructed on the training dataset. The analysis results can be found at **Supplementary: Feature analysis** in S1 File. The comparison test on dimension reduction was conducted with different numbers of principal components from 1 to 20 for all three pre-trained language models by 5-fold cross-validation through AutoGluon. Test results (in terms of *accuracy*) for AntiBERTy, ProtT5, and UniRep are listed at columns 2–4 in Table 1, respectively, which shows that the highest *accuracy* is 0.7911 for ProtT5 (with 4 and 5 principal components retained) and UniRep (with 19 principal components retained), while is 0.7458 for AntiBERTy with 2 principal components retained. The test results indicate that the best classification performance is obtained with the use of AntiBERTy for feature extraction and 2 principal components retained. Accordingly, AntiBERTy was finally chosen to extract the features which further to be compressed to 2 dimensions (which explains 27.1% of information on the original features) with the use of PCA.

**Table 1:** The 5-fold cross-validation results (*accuracy*) of the first 20 principal components respectively retained from the three features.

*n_components* *	AntiBERTy	ProtT5	UniRep
1	0.7119	0.6836	0.6780
2	**0.7458**	0.6893	0.6949
3	0.6780	0.6723	0.6949
4	0.7232	**0.7119**	0.7062
5	0.6949	**0.7119**	0.6836
6	0.6949	0.6836	0.6667
7	0.7062	0.7062	0.6610
8	0.6893	0.7119	0.6723
9	0.7062	0.6949	0.6667
10	0.7119	0.7062	0.6667
11	0.7119	0.6667	0.7006
12	0.7288	0.6667	0.6949
13	0.7288	0.6780	0.6836
14	0.7288	0.6780	0.6836
15	0.7345	0.6780	0.6949
16	0.7232	0.6893	0.6893
17	0.7345	0.7006	0.7006
18	0.7288	0.7062	0.6893
19	0.7175	0.6780	**0.7119**
20	0.7345	0.6780	0.6893

*The number of feature dimensions after dimension reduction using PCA.

### Model performance evaluation

The 5-fold cross-validation was chosen to construct the prediction model and evaluate the performance of the constructed model. The performance was further compared with PITHA on an independent test dataset. All test results are listed in Table 2, in which column 2 lists the 5-fold cross-validation result in terms of *ACC*; columns 3 to 9 show the comparison indexes used in this study. The verification result of 5-fold cross-validation shows that AbImmPred achieves 0.7458 in terms of *ACC*, which indicates the effectiveness of the constructed model.

**Table 2:** The 5-fold cross-validation results (*ACC*) on the training dataset and independent test results of AbImmPred and PITHA.

Method	Validation Results (*ACC*)	Independent Test Results						
		*ACC*	*Rec*	*Pre*	*F1*	*MCC*	*AUROC*	*AUPRC*
**PITHA**	-	0.6364	0.6875	**0.7857**	0.7333	**0.1736**	0.5938	0.7366
**AbImmPred**	0.7458	**0.7273**	**0.9375**	0.7500	**0.8333**	0.1614	**0.7813**	**0.9266**

**Fig 2:**
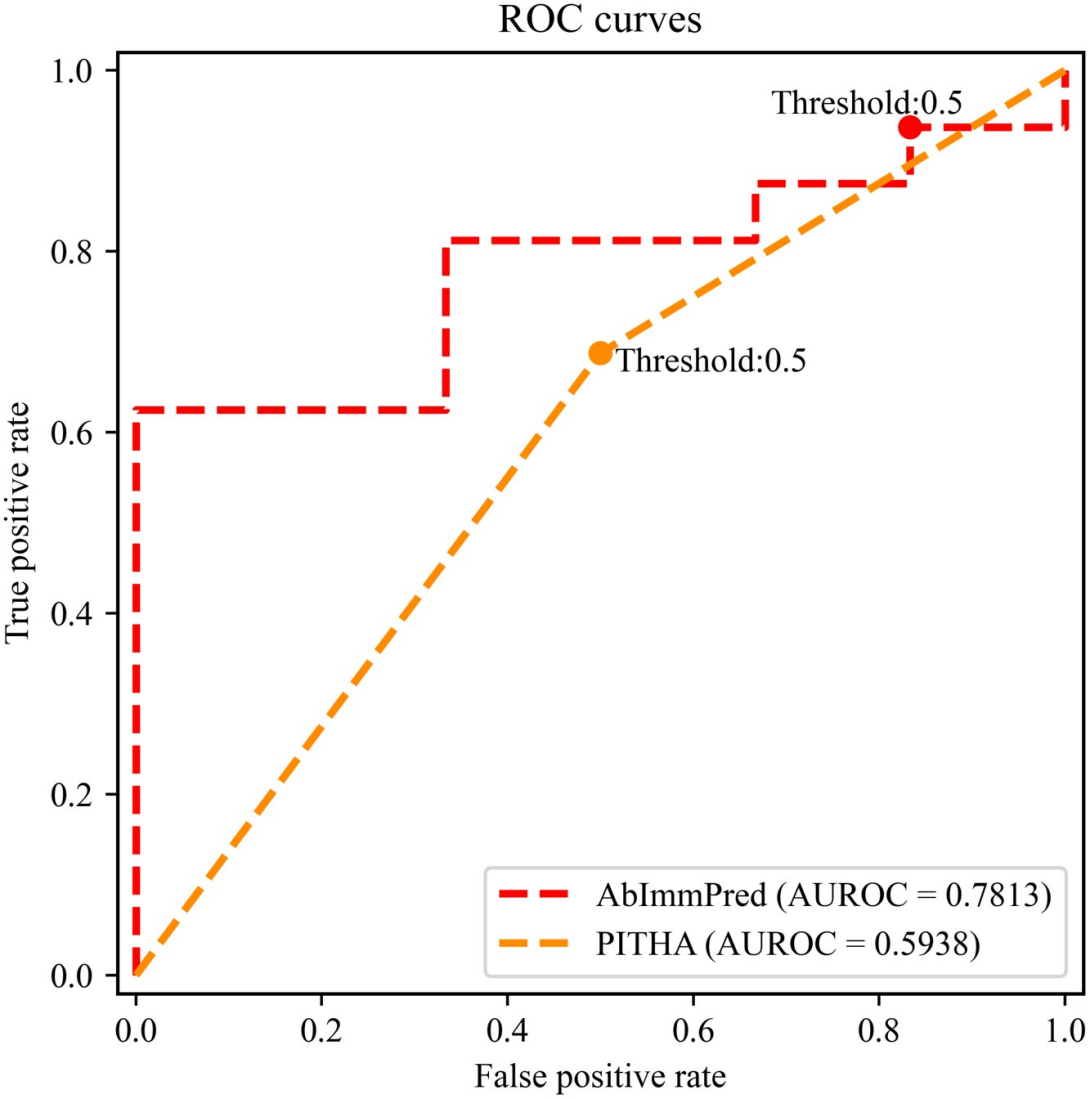
The ROC curves of AbImmPred and PITHA on the independent test dataset. With *FPR* as X-axis and *TPR* as Y-axis, the ROC curves are drawn, and the areas under the curves of AbImmPred and PITHA were marked as 0.7813 and 0.5938, respectively.

**Fig 3:**
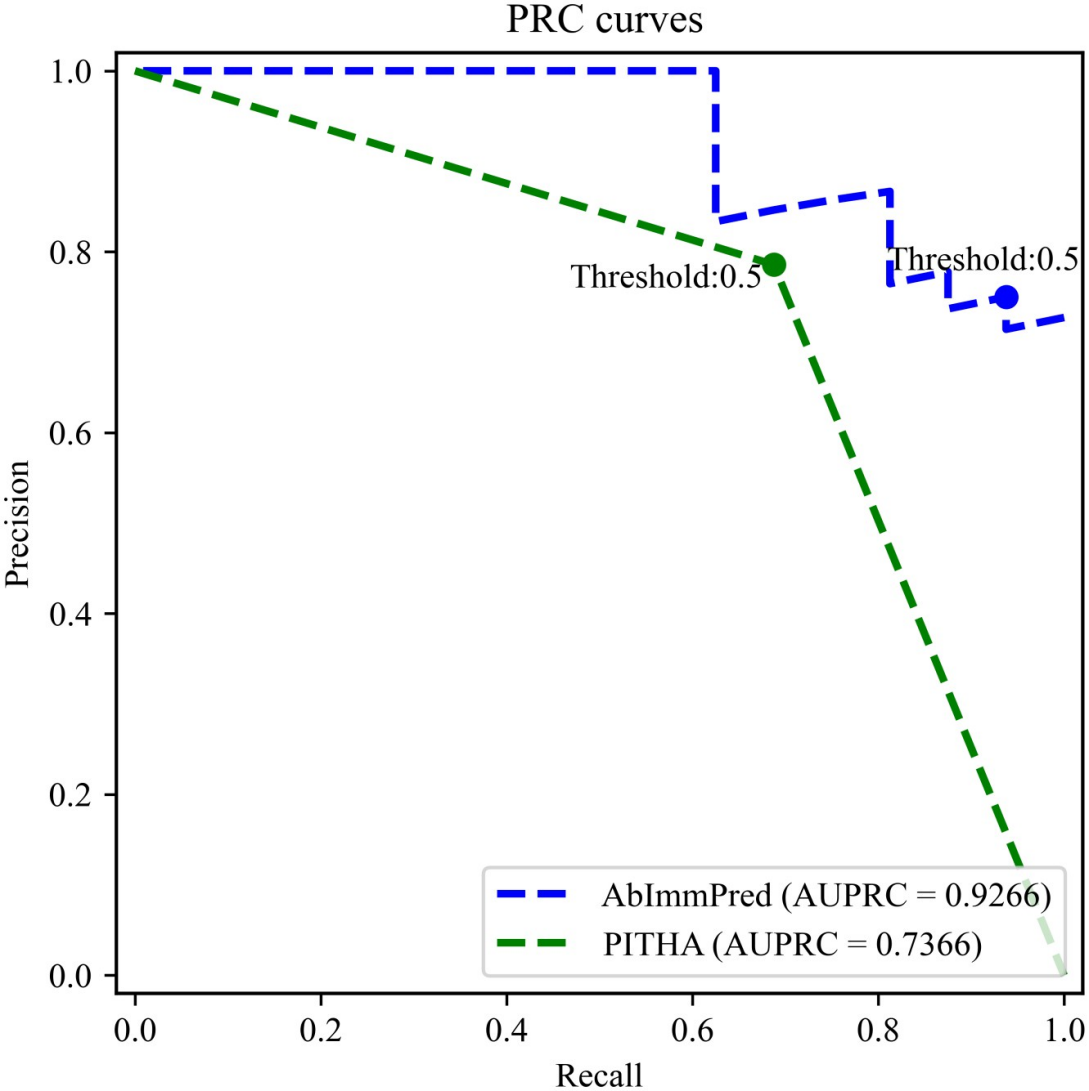
The PRC curves of AbImmPred and PITHA on the independent test dataset. With *Recall* as X-axis and *Precision* as Y-axis, the PRC curves are drawn, and the areas under the curves of AbImmPred and PITHA were marked as 0.9266 and 0.7366, respectively.

Test results on the independent test dataset show that the proposed method achieves 0.7273, 0.9375, 0.7500, 0.8333, and 0.1614 in terms of *ACC*, *Rec*, *Pre*, *F1*, and *MCC*, respectively. Among these evaluation indicators, the *ACC*, *Rec*, and *F1* are higher than that of the comparison method, PITHA, and outperform PITHA with 9.09%, 25.00%, and 1.00% respectively. In addition, the ROC curves and PRC curves of AbImmPred and PITHA are respectively drawn in Figs 2 and 3. Although PITHA’s web server only provides binary outputs, the two-step ROC curve and PRC curve based on its predictions could also be plotted for the purpose of clearly explaining the reported threshold-specific measures. The corresponding *AUROC* scores and *AUPRC* scores of AbImmPred and PITHA are also listed at columns 8 and 9 in Table 2. The results show that AbImmPred performs much better than PITHA.

Out of the 22 samples in the test dataset, 16 samples are positive and 6 are negative. Considering the imbalance problem of the dataset, random baseline (predicting all samples to be positive) was introduced to further compare the performance. The *ACC*, *Pre*, and *AUPRC* are all 72.7% for the random baseline. Compared to the random baseline, AbImmPred performs equally in terms of *ACC*, while much better in terms of *Pre* (0.7500) and *AUPRC* (0.9266 v.s. 0.7273, ~20% increase).

The 5-fold cross-validation result and independent test results indicate that AbImmPred has excellent predictive performance and good generalization ability. Especially in terms of *AUROC* score which is a more reliable index for measuring the performance, as the *AUPRC* is highly sensitive to class imbalance [34].

### Methods implementation and code availability

AbImmPred was implemented in Python 3.9.16, the PCA algorithm was borrowed from the Python based scikit-learn (version 1.0.2) package, and the version of AutoGluon is 0.5.2 We provide the online server for AbImmPred through https://www.genscript.com/tools/antibody-immunogenicity.

## Discussion

In this work, AbImmPred, a machine learning-based immunogenicity prediction model was proposed in which the feature representation of variable region sequences of therapeutic antibodies was extracted by the AntiBERTy pre-trained antibody language model; and AbImmPred was trained with an automatic machine learning framework, AutoGluon. AbImmPred can potentially be applied to the early screening stage of drug development in the biopharmaceutical industry.

There exist several obvious advantages of AbImmPred, which are listed as follows: **First**, the feature extraction process was simplified without sacrificing the representation ability of the original data by using pre-trained model to extract features for training. Not only that, the prediction performance of the constructed model could also be improved. As a contrast, the comparison method, PITHA, uses three-dimensional crystal structure or the modelled structure to calculate the structure-based features [22], which is a complex and difficult process. For a given antibody without its crystal structure, PITHA uses ABodyBuilder [35] for getting the 3D model first, then extracts the cavity volume at the CDR region and hydrophobicity of the CDR-H3 loop features, which is cumbersome and time-consuming. Besides that, the structural prediction and other operations could bring extra errors and further affect the *accuracy* of the final prediction results. The higher prediction *accuracy* of AbImmPred demonstrates the effectiveness of the pre-trained antibody language model in characterizing amino acid sequences and predicting the immunogenicity of therapeutic antibodies. **Second**, a larger dataset was used in AbImmPred, which helps to improve the prediction performance. There were 177 training samples used in AbImmPred, while only 52 samples (29 samples with crystal structure and 23 samples with modelled structure) in PITHA. Comparison results indicate that AbImmPred outer performs PITHA in terms of the important indicators, which provides evidence that a larger dataset is critical in machine learning. **Third**, the advanced AutoML package, AutoGluon, was used in AbImmPred, which trains multiple different machine learning models simultaneously (13 popular models and 1 weighted ensemble model) and optimizes the corresponding parameters automatically. Based on the validation score, AutoGluon automatically determines the best model. In this study, the predictive performance of each individual model and the weighted ensemble model in AutoGluon were also compared (please refer to **Supplementary: Performance of models in AutoGluon** in S1 File for details).

Despite the advantages mentioned above, the proposed method still has some limitations which could be further improved. **First**, even larger dataset was used in AbImmPred compared to PITHA, while the scale of the dataset is still small which should be further accumulated. **Second**, only the variable region of antibody was used in prediction under the consideration that therapeutic antibodies mainly bind to target molecules in the variable region to exert their effects. However, the constant region of antibody may have some potential functions for the whole antibody, e.g., the constant region may have a key role in maintaining the whole structure, in keeping the stability of the antibody, even in the binding process and other related functions. With the constant region sequences added, more potential immunogenicity related information could be involved in the prediction process, which will be considered in the future work. **Third**, the AntiBERTy model was used in this work as feature extractor, which is only the preliminary option. However, AntiBERTy model as well as other pre-trained models could be further fine-tuned in dealing with specific problems, including antibody immunogenicity prediction as higher-level usage in subsequent optimizations. **Fourth**, AbImmPred provides qualitative relationship between the antibody sequences and its immunogenicity (only high or low) because a classification problem was considered. While the quantitative relationship is more precious in the development of antibody therapeutics, which can capture the relative relationship and degree of difference between different samples, provide more information and details, and further help us to better understand and evaluate the immunogenicity level of the target antibodies. Quantitative analysis of immunogenicity can be achieved in the further work with sufficient training data accumulated by using machine learning or deep learning method which treats the immunogenicity problem as a regression problem. **Finally**, there exists a scalability problem of AbImmPred: theoretically, any antibody sequence even any amino acid sequence (such as peptides, proteins) can be input, while the current version of the web tool we provide can only support the monoclonal antibody sequence as input. The more types of antibodies such as nanobody and bi-specific antibody would be supported in the later version of the service with the accumulation of the data. The AbImmPred server provides an in-sequence-mode for the submitted query of an individual user, while a parallel-mode for different users. However, the local stand-alone version of AbImmPred could provide in-batch-mode service without too much computing resource and time added for any individual user as requested.

## Materials and methods

### Data collection

In this work, a total of 199 approved therapeutic antibodies were collected as training and testing datasets. The corresponding immunogenicity data were collected from the FDA website (https://www.accessdata.fda.gov/scripts/cder/daf/) and the corresponding variable region sequences of these therapeutic antibodies were obtained from the United States Patent and Trademark Office (USPTO, patft.uspto.gov) and/or the KEGG drug database (https://www.genome.jp/kegg/drug). The dataset was divided into positive (high immunogenicity) and negative (low immunogenicity) samples according to the number of patients with antigen-antibody reaction (AAR) in clinical treatment with a threshold of 2%, in which the samples with higher than 2% AAR are defined as high immunogenicity and others are low. Accordingly, 130 of the 199 antibodies are considered as high immunogenic and others are low. In addition, the dataset was split into training dataset (which contains 177 samples) and testing dataset (which contains 22 samples) for model construction and independent testing. The testing dataset was chosen in accordance with the dataset in Liang’s work [22] for a fair comparison, which has no overlap with the training dataset. The samples in test set are all humanized or fully humanized antibody sequences, while in training dataset are humanized, fully humanized, mouse, and chimeric antibodies. Dataset composition information is summarized in Table 3. The specific information of all therapeutic antibodies and their immunogenicity information in the dataset are shown in S1 and S2 Tables. The variable region sequences of therapeutic antibody samples are shown in S3 and S4 Tables.

**Table 3:** Dataset composition.

Dataset	Positive Samples	Negative Samples	Total
**Training Dataset**	114	63	177
**Independent Test Dataset**	16	6	22
**Total**	130	69	199

### Feature extraction

Feature extraction is an important step in machine learning method and has a direct influence on the final prediction/classification results. Recently, several language models-based pre-trained models for amino acid sequences features extraction were proposed, such as the most advanced transformer models-based antibody language model in the latest NLP research, AntiBERTy [27]; the protein language model, ProtT5-XL-UniRef50 [24]; recursive neural networks (RNN)-based UniRep [26]. In this study, AntiBERTy, ProtT5-XL-UniRef50 (ProtT5), and UniRep are used for testing and comparison.

According to the comparison results (please refer to **Supplementary: Feature analysis** in S1 File for details), AntiBERTy was finally chosen as the feature extraction method (please refer to **Supplementary: Feature extraction** in S1 File for details of feature extraction process of ProtT5 and UniRep). The features for heavy and light chains in variable regions were extracted separately but in a similar way using AntiBERTy. AntiBERTy uses the mask language model task to train a BERT model on a dataset containing 558M natural antibody sequences. The network parameters of AntiBERTy were set as follows: number of layers is set to 8 and with 8 attention heads for each layer, hidden dimension is set to 512, and the feedforward dimension is set to 2048. AntiBERTy maps discrete input data (antibody sequence) to a continuous word vector representation through an embedding layer, and the output of the embedding layer can be treated as feature to be input to subsequent models. The embedding of last layer of the encoder in AntiBERTy was extracted with heavy/light chain amino acid sequence as input.

In the embedding, each amino acid corresponds to a 512-dimensional vector, $V_{\begin{array}{c} aa_{i}\\ i=1,2,\cdots ,L \end{array}}$, expressed as

$$V_{\begin{array}{c} aa_{i}\\ i=1,2,\cdots ,L \end{array}}=[X_{aa_{i},1},\;X_{aa_{i},2},\cdots ,X_{aa_{i},n}]$$ (1)

where *n* = 512 represents the dimension of the embedding, *aa*_*i*_ represents the *i*-th amino acid in the sequence. Accordingly, the feature of the amino acid sequence can be expressed as a two-dimensional matrix with L*512 dimensions, marked as *F*_*H*_ for heavy chain, *F*_*L*_ for light chain,

$$F_{H}/F_{L}=\left[\begin{array}{c} V_{aa_{1}}\\ V_{aa_{2}}\\ \vdots \\ V_{aa_{L}} \end{array}\right]=\left[\begin{array}{c} X_{aa_{1},1},\;X_{aa_{1,}2},\;\cdots ,X_{aa_{1},n}\\ X_{aa_{2},1},\;X_{aa_{2},2},\;\cdots ,X_{aa_{2},n}\\ \vdots \\ X_{aa_{L},1},\;X_{aa_{L},2},\;\cdots ,X_{aa_{L},n} \end{array}\right]$$ (2)

where *L* is the length of the sequence. For getting a one-dimensional feature vector and making those elements in Eq (2) a comprehensive representation, *F*_*H*_ and *F*_*L*_ were compressed as *F*_*H*1_ and *F*_*L*1_ by columns as expressed in Eq (3):

$$F_{H1}/F_{L1}\;=\left[\frac{\sum_{i=1}^{L}X_{aa_{i},1}}{L},\frac{\sum_{i=1}^{L}X_{aa_{i},2}}{L},\cdots ,\frac{\sum_{i=1}^{L}X_{aa_{i},n}}{L}\right]$$ (3)

Then the feature vectors *F*_*H*1_ and *F*_*L*1_ were sequentially connected to obtain a 1024-dimension feature vector *F*_*H*+*L*_ as expressed in Eq (4).


$$F_{H+L}=[F_{H1},F_{L1}]$$ (4)

### Feature compression

In the training process of machine learning, more features do not always mean better learning performance. Redundant and irrelevant features will increase the difficulty in learning the relationship between features and objective values. In addition, more features mean more computational resources are needed. How to reduce the redundant and unnecessary even noise information in the high-dimension features without sacrificing the main information, comes to be an important problem in pre-processing of machine learning [36, 37].

In this study, principal component analysis (PCA) [38] was used for compressing the dimension of the extracted features, which is a commonly used feature dimension reduction method. The original feature obtained from the output of the embedding layer of AntiBERTy held a high-dimensionality compared to the training sample size, where redundant and noisy information always exists. The correlation between features could be considerably reduced by the dimension reduction operation with PCA. In addition, the overfitting problem could be avoided to a certain degree. On the basis of the maximum variance theory, PCA maps the original data (*n*-dimension) to a low dimension (*k*-dimension, *k*<*n*) through linear transformation, which makes the sample variance of each dimension as large as possible [38–40].

For implementing the feature dimension compression, a PCA model was first constructed on the training dataset using the scikit-learn [41] python package. The information proportions of each principal component feature were calculated for determining how many principal components should be used in the training process.

### Model construction

With the selected features at hand, the next step for machine learning is to construct the model. In this study, AutoGluon [42] was chosen to construct the prediction model, which is a new automated machine learning (AutoML) framework developed by Erickson et al. AutoGluon can automatically search for the best model architecture based on 13 basic machine learning models, including CatBoost [43], LightGBM [44], XGBoost [45], Random Forest (RF) [46], Extra Trees [47], K-nearest Neighbor (KNN) [48], Neural Networks [49, 50]. More detailed information of the models in AutoGluon can be found at **Supplementary: The basic machine learning models used in AutoGluonin** in the S1 File.

Besides, AutoGluon uses a multi-layer stack integration strategy for getting a weighted ensemble model to improve prediction accuracy. AutoGluon trains multiple different machine learning models which are then evaluated, and the best model can be selected according to the performance (e.g., *accuracy*) on the validation set. The weighted ensemble model in AutoGluon combines multiple basic models, which connects the outputs of above-mentioned models (base model) and the original features as input. Different weights are assigned to all base models based on its performance on the training dataset (better performance with higher weight). With the use of weighted integration, AutoGluon maximizes the advantages of each model and enhances the robustness and generalization ability of the ensemble model.

AutoGluon can automatically identify the type of prediction task (e.g., ‘binary’, ‘multiclass’, ‘regression’) according to the input labels, and the evaluation metric (*accuracy* by default in this study since the task is a binary classification problem) is then automatically selected on the basis of the type of the prediction task. Accordingly, only two parameters ‘*presets’* and ‘*num_bag_folds*’ were set to ‘best_quality’ and ‘5’, respectively, all other parameters were set to default in this study. The default hyperparameter values of machine learning models in AutoGluon were chosen at priori and can be found at github.com/awslabs/autogluon.

### Measurement indexes of different models

In this work, the 5-fold cross-validation on the training dataset was used to evaluate and select the model. The predictive performance of the final constructed model was further evaluated on an independent test dataset. Five evaluation indicators were used for measuring the performance of the model, which are *recall* (*Rec*), *precision* (*Pre*), *F1-score* (*F1*), *accuracy* (*ACC*) and *Matthew’s correlation coefficient* (*MCC*). These indicators are calculated by the following formula:

$$Rec=\;\frac{TP}{TP+FN}$$ (5)

$$Pre=\;\frac{TP}{TP+FP}$$ (6)

$$F1=2\times \frac{Rec\times Pre}{Rec\times Pre}$$ (7)

$$ACC=\frac{TP+TN}{TP+TN+FP+FN}$$ (8)

$$MCC=\;\frac{TP\times TN-FP\times FN}{\sqrt{\left(TP+FP\right)\left(TP+FN\right)\left(TN+FP\right)\left(TN+FN\right)}}$$ (9)

where *TP* represents the number of correctly classified positive samples, *TN* represents the number of correctly classified negative samples, *FP* represents the number of incorrectly classified positive samples, and *FN* represents the number of incorrectly classified negative samples.


$$FPR=\;\frac{FP}{FP+TN}$$ (10)

$$TPR=\;\frac{TP}{TP+FN}$$ (11)

In addition, the receiver operating characteristic curve (ROC curve) and Precision-Recall curve (PRC curve) were also drawn. The X-axis of ROC curve was false positive rate (*FPR*) and the Y-axis was true positive rate (*TPR*); the X-axis of *PRC* curve was *Rec* and the Y-axis was *Pre*. The area under the ROC curve (*AUROC* score) and PRC curve (*AUPRC* score) were accordingly calculated to further evaluate the performance of the model.

### Experimental settings for computational immunogenicity prediction

AbImmPred was implemented in Python 3.9.16 and ran in an Intel(R) Xeon(R) Bronze 3206R machine with 256 GB RAM, 1.90 GHz CPU, Nvidia A100 GPU, and 64-bit Ubuntu Sever 20.04 operating system. The version of AntiBERTy model is 0.1.3, which uses the [embed] function to generate sequence embeddings. The PCA algorithm was borrowed from the Python based scikit-learn (version 1.0.2) package, in which the parameters were set according to the prediction results after feature dimensionality reduction: *n_components* = 2, all other parameters were set to default. The version of AutoGluon is 0.5.2, in which the parameters were set: *presets* = ’best_quality’, *num_bag_folds* = 5, all other parameters were set to default.

## Supporting information

S1 File Supplementary material for AbImmPred: An immunogenicity prediction method for therapeutic antibodies using AntiBERTy-based sequence features. (DOCX)

S1 Table The names and immunogenicity values of 177 therapeutic antibody samples in the training dataset. (DOCX)

S2 Table The names and immunogenicity values of 22 therapeutic antibody samples in the independent test dataset. (DOCX)

S3 Table The variable region sequences of 177 therapeutic antibody samples in the training dataset. (XLSX)

S4 Table The variable region sequences of 22 therapeutic antibody samples in the independent test dataset. (XLSX)

## References

[1] Lagassé HAD, Alexaki A, Simhadri VL, Katagiri NH, Jankowski W, Sauna ZE, et al. Recent advances in (therapeutic protein) drug development. F1000Res. 2017; 6. doi: 10.12688/f1000research.9970.1 28232867 PMC5302153

[2] Lu RM, Hwang YC, Liu IJ, Lee CC, Tsai HZ, Li HJ, et al. Development of therapeutic antibodies for the treatment of diseases. J Biomed Sci. 2020; 27(1):1–30. doi: 10.1186/s12929-019-0592-z 31894001 PMC6939334

[3] Beck A, Wurch T, Bailly C, Corvaia N. Strategies and challenges for the next generation of therapeutic antibodies. Nat Rev Immunol. 2010; 10(5):345–352. doi: 10.1038/nri2747 20414207

[4] Baldo BA. Immune-and non-immune-mediated adverse effects of monoclonal antibody therapy: a survey of 110 approved antibodies. Antibodies. 2022; 11(1):17. doi: 10.3390/antib11010017 35323191 PMC8944650

[5] Baker M, Reynolds HM, Lumicisi B, Bryson CJ. Immunogenicity of protein therapeutics: the key causes, consequences and challenges. Self/nonself. 2010; 1(4):314–322. doi: 10.4161/self.1.4.13904 21487506 PMC3062386

[6] Kurki P, van Aerts L, Wolff-Holz E, Giezen T, Skibeli V, Weise M. Interchangeability of biosimilars: a European perspective. BioDrugs. 2017; 31(2):83–91. doi: 10.1007/s40259-017-0210-0 28120313

[7] De Groot AS. Immunomics: discovering new targets for vaccines and therapeutics. Drug Discov Today. 2006; 11(5–6):203–209. doi: 10.1016/S1359-6446(05)03720-7 16580597

[8] Dingman R, Balu-Iyer SV. Immunogenicity of protein pharmaceuticals. J Pharm Sci. 2019; 108(5):1637–1654. doi: 10.1016/j.xphs.2018.12.014 30599169 PMC6720129

[9] Yin L, Chen X, Vicini P, Rup B, Hickling TP. Therapeutic outcomes, assessments, risk factors and mitigation efforts of immunogenicity of therapeutic protein products. Cell Immunol. 2015; 295(2):118–126. doi: 10.1016/j.cellimm.2015.03.002 25880103

[10] Karalis VD. From bioequivalence to biosimilarity: the rise of a novel regulatory framework. Drug Res. 2016; 66(01):1–6. doi: 10.1055/s-0035-1548911 25894088

[11] De Groot AS, Scott DW. Immunogenicity of protein therapeutics. Trends Immunol. 2007; 28(11):482–490. doi: 10.1016/j.it.2007.07.011 17964218

[12] Kuroda D, Tsumoto K. Engineering stability, viscosity, and immunogenicity of antibodies by computational design. J Pharm Sci. 2020, 109(5): 1631–1651. doi: 10.1016/j.xphs.2020.01.011 31958430

[13] Kazi A, Chuah C, Majeed ABA, Leow CH, Lim BH, Leow CY. Current progress of immunoinformatics approach harnessed for cellular-and antibody-dependent vaccine design. Pathog Glob Health. 2018; 112(3):123–131. doi: 10.1080/20477724.2018.1446773 29528265 PMC6056828

[14] Pratt KP. Anti-drug antibodies: emerging approaches to predict, reduce or reverse biotherapeutic immunogenicity. Antibodies. 2018; 7(2):19. doi: 10.3390/antib7020019 31544871 PMC6698869

[15] Doneva N, Doytchinova I, Dimitrov I. Predicting immunogenicity risk in biopharmaceuticals. Symmetry. 2021; 13(3):388. doi: 10.3390/sym13030388

[16] Oyarzún P, Ellis JJ, Bodén M, Kobe B. PREDIVAC: CD4+ T-cell epitope prediction for vaccine design that covers 95% of HLA class II DR protein diversity. BMC Bioinform. 2013; 14(1):1–11. doi: 10.1186/1471-2105-14-52 23409948 PMC3598884

[17] Bhasin M, Raghava GPS. Prediction of CTL epitopes using QM, SVM and ANN techniques. Vaccine. 2004; 22(23–24):3195–3204. doi: 10.1016/j.vaccine.2004.02.005 15297074

[18] Sweredoski MJ, Baldi P. PEPITO: improved discontinuous B-cell epitope prediction using multiple distance thresholds and half sphere exposure. Bioinform. 2008; 24(12):1459–1460. doi: 10.1093/bioinformatics/btn199 18443018

[19] Sweredoski MJ, Baldi P. COBEpro: a novel system for predicting continuous B-cell epitopes. Protein Eng Des Sel. 2009; 22(3):113–120. doi: 10.1093/protein/gzn075 19074155 PMC2644406

[20] Liang S, Zheng D, Standley DM, Yao B, Zacharias M, Zhang C. EPSVR and EPMeta: prediction of antigenic epitopes using support vector regression and multiple server results. BMC Bioinform. 2010; 11(1):1–6. doi: 10.1186/1471-2105-11-381 20637083 PMC2910724

[21] Mason DM, Friedensohn S, Weber CR, Jordi C, Wagner B, Meng SM, et al. Optimization of therapeutic antibodies by predicting antigen specificity from antibody sequence via deep learning. Nat Biomed Eng. 2021, 5(6): 600–612. doi: 10.1038/s41551-021-00699-9 33859386

[22] Liang S, Zhang C. Prediction of immunogenicity for humanized and full human therapeutic antibodies. PLoS One. 2020; 15(8):e0238150. doi: 10.1371/journal.pone.0238150 32866159 PMC7458303

[23] Rives A, Meier J, Sercu T, Goyal S, Lin Z, Liu J, et al. Biological structure and function emerge from scaling unsupervised learning to 250 million protein sequences. Proc Natl Acad Sci. 2021; 118(15):e2016239118. doi: 10.1073/pnas.2016239118 33876751 PMC8053943

[24] Elnaggar A, Heinzinger M, Dallago C, Rehawi G, Wang Y, Jones L, et al. Prottrans: Toward understanding the language of life through self-supervised learning. IEEE Trans Pattern Anal Mach Intell. 2021; 44(10):7112–7127. doi: 10.1109/TPAMI.2021.3095381 34232869

[25] Gane A, Bileschi ML, Dohan D, Speretta E, Héliou A, Meng-Papaxanthos L. et al. ProtNLM: Model-based Natural Language Protein Annotation. 2022.

[26] Alley EC, Khimulya G, Biswas S, AlQuraishi M, Church GM. Unified rational protein engineering with sequence-based deep representation learning. Nat Methods. 2019; 16(12):1315–1322. doi: 10.1038/s41592-019-0598-1 31636460 PMC7067682

[27] Ruffolo JA, Gray JJ, Sulam J. Deciphering antibody affinity maturation with language models and weakly supervised learning. arXiv preprint arXiv:2112.07782. 2021.

[28] Leem J, Mitchell LS, Farmery JHR, Barton J, Galson JD. Deciphering the language of antibodies using self-supervised learning. Patterns. 2022; 3(7):100513. doi: 10.1016/j.patter.2022.100513 35845836 PMC9278498

[29] Wang D, Ye F, Zhou H. On Pre-trained Language Models for Antibody. bioRxiv. 2023; 2023.01. 29.525793. doi: 10.1101/2023.01.29.525793

[30] Ofer D, Brandes N, Linial M. The language of proteins: NLP, machine learning & protein sequences. Comput Struct Biotechnol J. 2021; 19:1750–1758. doi: 10.1016/j.csbj.2021.03.022 33897979 PMC8050421

[31] Park M, Seo S, Park E, Kim J. EpiBERTope: a sequence-based pre-trained BERT model improves linear and structural epitope prediction by learning long-distance protein interactions effectively. bioRxiv. 2022; 2022.02. 27.481241. doi: 10.1101/2022.02.27.481241

[32] Wang F, Wang H, Wang L, Lu H, Qiu S, Zang T, et al. MHCRoBERTa: pan-specific peptide–MHC class I binding prediction through transfer learning with label-agnostic protein sequences. Brief Bioinform. 2022; 23(3):bbab595. doi: 10.1093/bib/bbab595 35443027

[33] Wong TT. Performance evaluation of classification algorithms by k-fold and leave-one-out cross validation. Pattern Recognit. 2015; 48(9):2839–2846. doi: 10.1016/j.patcog.2015.03.009

[34] Flach P, Kull M. Precision-recall-gain curves: PR analysis done right. Adv Neural Inf Process Syst, 2015, 28(1): 838–846.

[35] Leem J, Dunbar J, Georges G, Shi J, Deane CM. ABodyBuilder: Automated antibody structure prediction with data–driven accuracy estimation. MAbs. 2016; 8(7):1259–1268. doi: 10.1080/19420862.2016.1205773 27392298 PMC5058620

[36] Li M, Wang H, Yang L, Liang Y, Shang Z, Wan H. Fast hybrid dimensionality reduction method for classification based on feature selection and grouped feature extraction. Expert Syst Appl. 2020; 150:113277. doi: 10.1016/j.eswa.2020.113277

[37] Zebari R, Abdulazeez A, Zeebaree D, Zebari D, Saeed J. A comprehensive review of dimensionality reduction techniques for feature selection and feature extraction. J Appl Sci Technol Trends. 2020; 1(2):56–70. doi: 10.38094/jastt1224

[38] Karamizadeh S, Abdullah SM, Manaf AA, Zamani M, Hooman A. An overview of principal component analysis. J Signal Inf Process. 2013; 4(3B):173. doi: 10.4236/jsip.2013.43B031

[39] Jolliffe IT, Cadima J. Principal component analysis: a review and recent developments. Philos Trans A Math Phys Eng Sci. 2016; 374(2065):20150202. doi: 10.1098/rsta.2015.0202 26953178 PMC4792409

[40] Abdi H, Williams LJ. Principal component analysis. Wiley Interdiscip Rev Comput Stat. 2010; 2(4):433–459. doi: 10.1002/wics.101

[41] Pedregosa F, Varoquaux G, Gramfort A, Michel V, Thirion B, Grisel O, et al. Scikit-learn: Machine learning in Python. J Mach Learn Res. 2011, 12:2825–2830. doi: 10.1007/978-1-4842-4470-8_18

[42] Erickson N, Mueller J, Shirkov A, Zhang H, Larroy P, Li M, et al. Autogluon-tabular: Robust and accurate automl for structured data. arXiv preprint arXiv:2003.06505. 2020.

[43] Dorogush AV, Ershov V, Gulin A. CatBoost: gradient boosting with categorical features support. arXiv preprint arXiv:1810.11363. 2018.

[44] Ke G, Meng Q, Finley T, Wang T, Chen W, Ma W, et al. Lightgbm: A highly efficient gradient boosting decision tree. Adv Neural Inf Process Syst. 2017; 30:3149–3157.

[45] Chen T, Guestrin C. Xgboost: A scalable tree boosting system. Proceedings of the 22nd acm sigkdd international conference on knowledge discovery and data mining. New York (NY): Association for Computing Machinery; 2016.p.785-794.

[46] Biau G, Scornet E. A random forest guided tour. Test. 2016; 25:197–227. doi: 10.1007/s11749-016-0481-7

[47] Geurts P, Ernst D, Wehenkel L. Extremely randomized trees[J]. Mach Learn. 2006, 63: 3–42. doi: 10.1007/s10994-006-6226-1

[48] Zhang Z. Introduction to machine learning: k-nearest neighbors. Ann Transl Med. 2016, 4(11). doi: 10.21037/atm.2016.03.37 27386492 PMC4916348

[49] Howard J, Gugger S. Fastai: A layered API for deep learning. Inf. 2020; 11(2):108. doi: 10.3390/info11020108

[50] Stevens E, Antiga L, Viehmann T. Deep learning with PyTorch. New York (NY): Manning Publications; 2020.

