# Supplementary material for: AbImmPred: An immunogenicity prediction method for therapeutic antibodies using AntiBERTy-based sequence features
Source: PLoS One. 2024 Feb 23;19(2):e0296737. doi: 10.1371/journal.pone.0296737 (PMC10889861; doi:10.1371/journal.pone.0296737)
Supplement: S1 File — (DOCX) [file pone.0296737.s001.docx]

Supplementary Material for AbImmPred: An Immunogenicity Prediction Method for Therapeutic Antibodies Using AntiBERTy-based Sequence Features

Hong Wang^1^, Xiaohu Hao^1^, Yuzhuo He^1^ and Long Fan ^2*^

^1^ Production and R&D Center I of Life Science Services, GenScript Biotech Corporation, Nanjing, China

^2^ Production and R&D Center I of Life Science Services, GenScript (Shanghai) Biotech Co.,Ltd., Shanghai, China

* Corresponding author

E-mail: [leo.fan@genscript.com](mailto:leo.fan@genscript.com) (LF)

## Supplementary: The basic machine learning models used in AutoGluon

In this part, the basic machine learning models available in AutoGluon are introduced, including CatBoost, LightGBM, XGBoost, Random Forest (RF), Extra Trees, K-nearest Neighbor (KNN), and Neural Networks.

CatBoost, LightGBM, and XGBoost are machine learning models based on Gradient Boosting Decision Tree (GBDT). CatBoost utilizes multiple decision trees to build a robust predictive model. It incorporates a specialized treatment for class features known as Symmetric Tree-based Encoding, which enhances the accuracy of the model. CatBoost also features an adaptive learning rate adjustment strategy that automatically improves training results.

LightGBM utilizes a growth strategy known as "leaf-wise" to enhance model accuracy. This strategy selects nodes that lead to the quickest decrease in the loss function during splitting, enabling faster identification of the optimal split point. Moreover, LightGBM incorporates a histogram algorithm to accelerate the feature discretization process, resulting in improved training speed.

XGBoost introduces regularization terms based on the GBDT to prevent overfitting. It employs a unique split node selection strategy known as the Approximate Greedy Algorithm, which efficiently identifies the optimal split point. XGBoost also supports parallel training and prediction, making it well-suited for handling high-dimensional sparse data and large-scale datasets effectively.

RF is an ensemble learning model consisting of multiple decision trees that make predictions by voting or averaging. In AutoGluon, the RF model supports the use of two different splitting criteria: Entropy-based on information gain and Gini-based on the Gini index. RF reduces the variance of the model by introducing randomness, thereby reducing the risk of overfitting.

Extra Trees introduces more randomness on top of RF. When constructing a decision tree, Extra Trees not only randomly samples data, but also randomly selects each feature, randomly selects a partition threshold for a feature, and ultimately selects the best partition feature and threshold to build the decision tree. In AutoGluon, the Extra Trees model also supports two different splitting criteria: Entropy and Gini.

KNN is an instance-based learning method that calculates the distance between samples for classification or regression. In AutoGluon, the KNN model supports two different weight strategies: Uniform and Distance. The Uniform weighting strategy assigns equal weights to all nearest neighbor samples, while the Distance weighting strategy assigns weights based on the reciprocal of the distance.

AutoGluon supports training and prediction using two different neural network frameworks, FastAI (neural network with FastAI backend) and Torch (neural network implemented in Pytorch). Pytorch is a lower-level deep learning library that provides more flexibility and control over the neural network architecture and training process. It allows users to define custom neural network architectures and implement complex training algorithms. FastAI is an advanced deep learning library built on top of Pytorch, developed by Howard and Gugger. It provides a high-level API designed to simplify deep learning tasks. FastAI offers a range of tools for data preprocessing, model training, and visualization. It also supports pre-trained models and transfer learning, making it easier to apply deep learning to real-world problems.

## Supplementary: Feature extraction

In addition to AntiBERTy, the use of ProtT5 and UniRep for feature extraction is also tested in this study. ProtT5 is derived from the BERT model and trained in UniRef50 corpora. UniRep uses RNN to learn statistical representations of proteins from the UniRef50 corpora.

In ProtT5, the steps of feature extraction are basically the same as those of AntiBERTy model. The difference is that in the embedding of the last layer of the encoder in ProtT5, each amino acid corresponds to a 1024-dimensional vector. Similar to the splice operation when using AntiBERTy, the final feature of the given antibody would be a vector with 2048 dimensions.

UniRep contains three architectures (64, 256, and 1900 units respectively). The output of UniRep (with 1900 units) represents the whole input sequence by a vector with 1900 dimensions. Similar to the splice operation when using ProtT5, the final feature of the given antibody would be a vector with 3800 dimensions.

## Supplementary: Feature analysis

For determining which of the three features is the most suitable one for antibody immunogenicity prediction, feature analysis was performed by feature compression and selection.

Due to the high dimensionality of the obtained features, which easily causes the overfitting problem, PCA was hired to do the feature compression operation. By applying PCA to the extracted features, the original features are transferred to a new group of features (principal component). The information ratio of each principal component was calculated and depicted in **S1a-S1c Figs**. As shown in the figures, the first 20 principal components explain 65.6%, 79.4%, and 65.1% information of the entire features for AntiBERTy (**S1a Fig**), ProtT5 (**S1b Fig**), and UniRep (**S1c Fig**), respectively. Especially for the features extracted from AntiBERTy and ProtT5, the first one principal component accounts for 17.3% and 24.3% information of entire features, respectively, which gives indication of the choice of compressed features.

**S1 Fig. The principal component variance contribution rate.** With the number of principal components as the X-axis and the proportion of variance explained by the corresponding principal components as the Y-axis, the bar plots of the contribution rate of principal component variance of the features are drawn. **a:** Bar plot of the proportion of variance explained of the first 20 principal components of features extracted by AntiBERTy. **b:** Bar plot of the proportion of variance explained of the first 20 principal components of features extracted by ProtT5. **c:** Bar plot of the proportion of variance explained of the first 20 principal components of features extracted by UniRep.


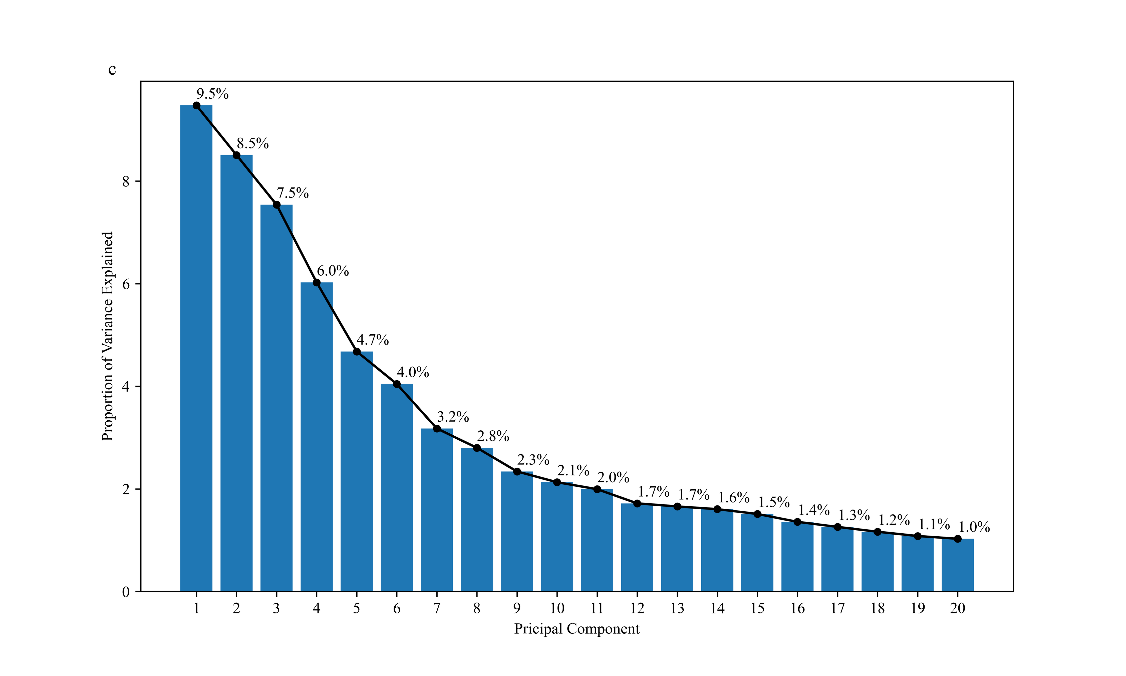

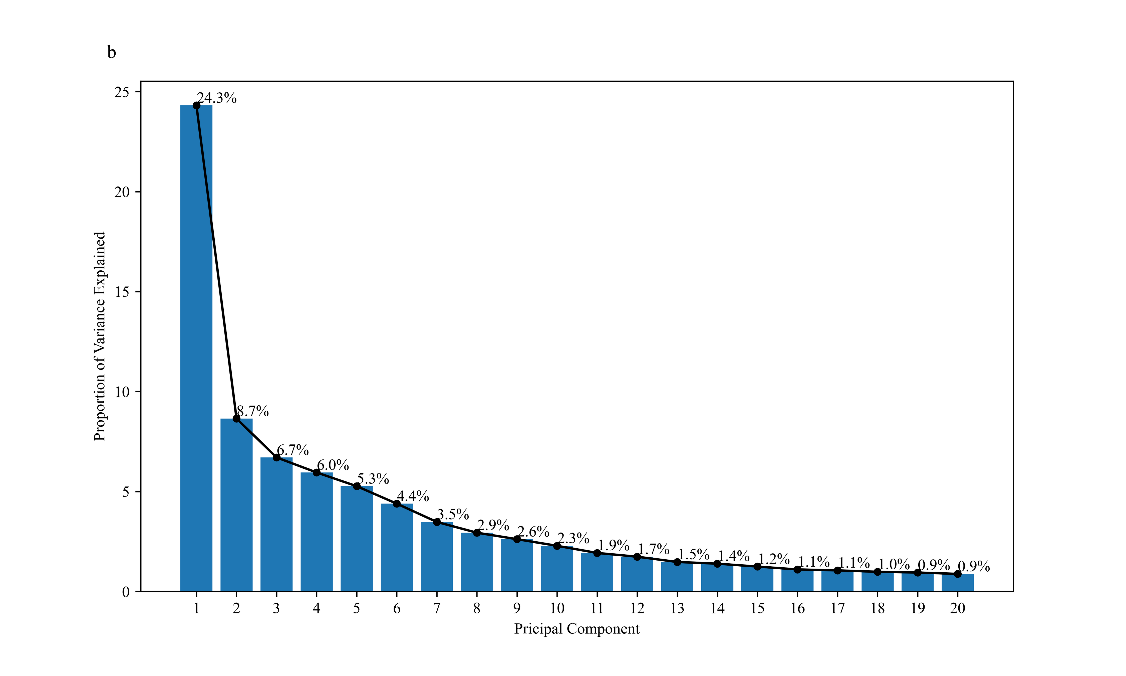

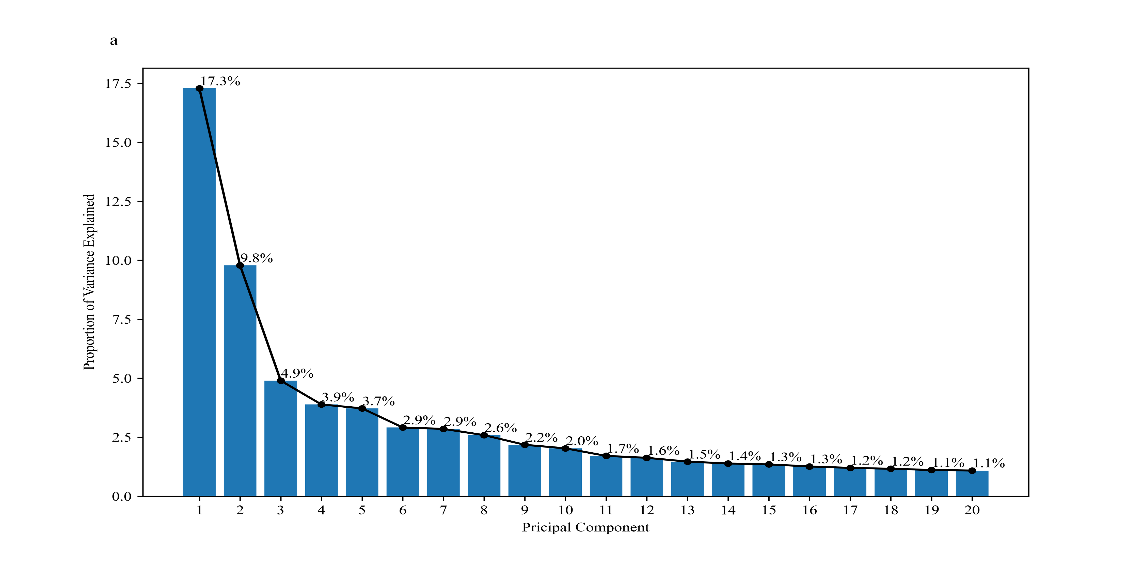


## Supplementary: Performance of models in AutoGluon

AutoGluon trains not only 13 popular machine learning models but also a weighted ensemble model. In order to further compare the predictive performance of all 13 independent models and the extra weighted ensemble models, AutoGluon was fitted by the three types of features (each of them was reduced to the optimal dimension, AntiBERTY: 2 dimensions, ProtT5: 4 dimensions, UniRep: 19 dimensions) respectively. The 5-fold cross-validation results of each model trained with features extracted by AntiBERTy, ProtT5, and UniRep are listed in **S5 Table**. Column 2 lists the names of each model, column 3 to 5 list the 5-fold cross-validation results in terms of *accuracy* which were obtained with the use of AntiBERTy, ProtT5, and UniRep for feature extraction, respectively. The validation results show that different sub-model in AutoGluon achieved the best performance (Neural Network (Torch) for the features extracted by UniRep, LightGBM (Large) for both features extracted by AntiBERTy and ProtT5). Meanwhile, the weighted ensemble model also and always achieved the best performance, which shows the weighted ensemble model has the potential in improving the prediction performance of independent machine learning models.

**S5 Table. The 5-fold cross-validation results (accuracy) of each model in AutoGluon trained with three kinds of features.**

| **No.** | **Models** | **AntiBERTy** | **ProtT5** | **UniRep** |
| --- | --- | --- | --- | --- |
| **1** | Weighted Ensemble Model | **0.7458** | **0.7119** | **0.7119** |
| **2** | LightGBM（Large） | 0.7288 | **0.7119** | 0.6610 |
| **3** | CatBoost | 0.7288 | 0.6836 | 0.6780 |
| **4** | LightGBM | 0.7232 | 0.6893 | 0.6893 |
| **5** | Neural Network（FastAI） | 0.7175 | 0.6554 | 0.6780 |
| **6** | XGBoost | 0.6893 | 0.6780 | 0.6723 |
| **7** | LightGBM (extra_trees) | 0.6836 | 0.6667 | 0.6780 |
| **8** | Neural Network（Torch） | 0.6441 | 0.6497 | **0.7119** |
| **9** | Extra Trees (Gini) | 0.6441 | 0.5706 | 0.5819 |
| **10** | KNN (Uniform) | 0.6271 | 0.4859 | 0.5819 |
| **11** | Extra Trees (Entropy) | 0.6215 | 0.5819 | 0.6158 |
| **12** | Random Forest (Gini) | 0.6215 | 0.5480 | 0.5650 |
| **13** | KNN (Distance) | 0.6102 | 0.4972 | 0.5763 |
| **14** | Random Forest (Entropy) | 0.6102 | 0.5650 | 0.6215 |
