# Supplementary material for: AbImmPred: An immunogenicity prediction method for therapeutic antibodies using AntiBERTy-based sequence features
Source: PLoS One. 2024 Feb 23;19(2):e0296737. doi: 10.1371/journal.pone.0296737 (PMC10889861; doi:10.1371/journal.pone.0296737)
Supplement: S1 Table — (DOCX) [file pone.0296737.s002.docx]

**S1 Table**. **The names and immunogenicity values of 177 therapeutic antibody samples in the training dataset.**

| **No.** | **Therapeutic antibody** | **Immunogenicity value (%)** | **Immunogenicity label*** |
| --- | --- | --- | --- |
| **1** | Blinatumomab | 1.4 | 0 |
| **2** | Basiliximab | 1.5 | 0 |
| **3** | Bavituximab | 58 | 1 |
| **4** | Rituximab | 11 | 1 |
| **5** | Siltuximab | 0.2 | 0 |
| **6** | Alemtuzumab | 5.1 | 1 |
| **7** | Atezolizumab | 28.9 | 1 |
| **8** | Bevacizumab | 0.3 | 0 |
| **9** | Certolizumab | 8 | 1 |
| **10** | Daclizumab | 19 | 1 |
| **11** | Farletuzumab | 8.7 | 1 |
| **12** | Matuzumab | 10 | 1 |
| **13** | Omalizumab | 0 | 0 |
| **14** | Pembrolizumab | 1.7 | 0 |
| **15** | Pertuzumab | 2.8 | 1 |
| **16** | Ranibizumab | 3.5 | 1 |
| **17** | Trastuzumab | 8.1 | 1 |
| **18** | Adalimumab | 14 | 1 |
| **19** | Durvalumab | 3.3 | 1 |
| **20** | Ipilimumab | 2 | 1 |
| **21** | Necitumumab | 4.1 | 1 |
| **22** | Nivolumab | 7.7 | 1 |
| **23** | Ofatumumab | 0 | 0 |
| **24** | Ramucirumab | 4.1 | 1 |
| **25** | Zalutumumab | 0 | 0 |
| **26** | Moxetumomab | 59 | 1 |
| **27** | Abagovomab | 68.1 | 1 |
| **28** | Begelomab | 28 | 1 |
| **29** | PankoMab | 25 | 1 |
| **30** | Infliximab | 30.5 | 1 |
| **31** | Eculizumab | 2 | 1 |
| **32** | Natalizumab | 9 | 1 |
| **33** | Ustekinumab | 4 | 1 |
| **34** | Avelumab | 5 | 1 |
| **35** | Canakinumab | 0 | 0 |
| **36** | Panitumumab | 2.8 | 1 |
| **37** | Brentuximab | 35 | 1 |
| **38** | Dinutuximab | 17 | 1 |
| **39** | Polatuzumab | 2.6 | 1 |
| **40** | Concizumab | 17 | 1 |
| **41** | Gevokizumab | 37 | 1 |
| **42** | Lebrikizumab | 30 | 1 |
| **43** | Clivatuzumab | 10 | 1 |
| **44** | Obexelimab | 17.5 | 1 |
| **45** | Talacotuzumab | 17.5 | 1 |
| **46** | Vatelizumab | 2.6 | 1 |
| **47** | Erenumab | 2.4 | 1 |
| **48** | Tezepelumab | 2.1 | 1 |
| **49** | Tralokinumab | 0.5 | 0 |
| **50** | Berlimatoxumab | 0 | 0 |
| **51** | Coltuximab | 33 | 1 |
| **52** | Ficlatuzumab | 0 | 0 |
| **53** | Ocaratuzumab | 1 | 0 |
| **54** | Ozanezumab | 2.5 | 1 |
| **55** | Temelimab | 0 | 0 |
| **56** | Veltuzumab | 19.5 | 1 |
| **57** | Navicixizumab | 29 | 1 |
| **58** | Palivizumab | 1.1 | 0 |
| **59** | Naptumomab | 72.5 | 1 |
| **60** | Xentuzumab | 2.3 | 1 |
| **61** | Andecaliximab | 21.2 | 1 |
| **62** | Ibalizumab | 15 | 1 |
| **63** | Ublituximab | 0 | 0 |
| **64** | Zolbetuximab | 0 | 0 |
| **65** | Fontolizumab | 7.8 | 1 |
| **66** | Futuximab | 0 | 0 |
| **67** | Labetuzumab | 0 | 0 |
| **68** | Modotuximab | 0 | 0 |
| **69** | Tomuzotuximab | 12.5 | 1 |
| **70** | Tigatuzumab | 0 | 0 |
| **71** | Gremubamab | 2.5 | 1 |
| **72** | Inclacumab | 3.6 | 1 |
| **73** | Ponezumab | 0 | 0 |
| **74** | Setrusumab | 0 | 0 |
| **75** | Rovalpituzumab | 0 | 0 |
| **76** | Fulranumab | 2.9 | 1 |
| **77** | Guselkumab | 6 | 1 |
| **78** | Galcanezumab | 8.7 | 1 |
| **79** | Netakimab | 0 | 0 |
| **80** | Risankizumab | 24 | 1 |
| **81** | Romosozumab | 18.1 | 1 |
| **82** | Toripalimab | 1 | 0 |
| **83** | Racotumomab | 85 | 1 |
| **84** | Zolimomab | 85.7 | 1 |
| **85** | Aducanumab | 5 | 1 |
| **86** | Anifrolumab | 3.3 | 1 |
| **87** | Bococizumab | 25.5 | 1 |
| **88** | Bimekizumab | 12.8 | 1 |
| **89** | Birtamimab | 0 | 0 |
| **90** | Brazikumab | 2.5 | 1 |
| **91** | Clazakizumab | 1.8 | 0 |
| **92** | Crenezumab | 0 | 0 |
| **93** | Depatuxizumab | 5 | 1 |
| **94** | Etrolizumab | 5 | 1 |
| **95** | Ganitumab | 5.2 | 1 |
| **96** | Glembatumumab | 3.8 | 1 |
| **97** | Ligelizumab | 6.3 | 1 |
| **98** | Motavizumab | 1.5 | 0 |
| **99** | Nirsevimab | 28 | 1 |
| **100** | Olokizumab | 9.8 | 1 |
| **101** | Onartuzumab | 11.9 | 1 |
| **102** | Pamrevlumab | 0 | 0 |
| **103** | Roledumab | 0 | 0 |
| **104** | Sirukumab | 2.9 | 1 |
| **105** | Solanezumab | 3.5 | 1 |
| **106** | Suptavumab | 5 | 1 |
| **107** | Sutimlimab | 8 | 1 |
| **108** | Tabalumab | 4.4 | 1 |
| **109** | Ticilimumab | 2 | 1 |
| **110** | Abrilumab | 0.4 | 0 |
| **111** | Amatuximab | 29.2 | 1 |
| **112** | Bleselumab | 2.2 | 1 |
| **113** | Cendakimab | 3 | 1 |
| **114** | Cinpanemab | 0 | 0 |
| **115** | Conatumumab | 0 | 0 |
| **116** | Dacetuzumab | 0 | 0 |
| **117** | Dalotuzumab | 0 | 0 |
| **118** | Dapirolizumab | 8.9 | 1 |
| **119** | Disitamab | 58.6 | 1 |
| **120** | Dusigitumab | 0 | 0 |
| **121** | Eldelumab | 0 | 0 |
| **122** | Emibetuzumab | 0 | 0 |
| **123** | Enokizumab | 9 | 1 |
| **124** | Frovocimab | 6.5 | 1 |
| **125** | Galiximab | 0 | 0 |
| **126** | Gancotamab | 2.9 | 1 |
| **127** | Gedivumab | 1 | 0 |
| **128** | Gosuranemab | 6.2 | 1 |
| **129** | Icrucumab | 0 | 0 |
| **130** | Imalumab | 7.4 | 1 |
| **131** | Indusatumab | 0 | 0 |
| **132** | Etaracizumab | 0 | 0 |
| **133** | Iscalimab | 1.8 | 0 |
| **134** | Levilimab | 0 | 0 |
| **135** | Lifastuzumab | 18 | 1 |
| **136** | Lintuzumab | 0 | 0 |
| **137** | Lorvotuzumab | 0 | 0 |
| **138** | Lutikizumab | 16.8 | 1 |
| **139** | Mavrilimumab | 3.1 | 1 |
| **140** | Namilumab | 0.9 | 0 |
| **141** | Ontuxizumab | 4.5 | 1 |
| **142** | Opicinumab | 3.6 | 1 |
| **143** | Otlertuzumab | 5 | 1 |
| **144** | Parsatuzumab | 4.7 | 1 |
| **145** | Pateclizumab | 4.7 | 1 |
| **146** | Pinatuzumab | 1.4 | 0 |
| **147** | Prasinezumab | 0 | 0 |
| **148** | Prezalumab | 0 | 0 |
| **149** | Refanezumab | 9.4 | 1 |
| **150** | Robatumumab | 0 | 0 |
| **151** | Sifalimumab | 0 | 0 |
| **152** | Suvratoxumab | 3 | 1 |
| **153** | Tarextumab | 26 | 1 |
| **154** | Tovetumab | 2.9 | 1 |
| **155** | Mogamulizumab | 4.2 | 1 |
| **156** | Varlilumab | 0 | 0 |
| **157** | Utomilumab | 40.7 | 1 |
| **158** | Domagrozumab | 9.4 | 1 |
| **159** | Gantenerumab | 0 | 0 |
| **160** | Lanadelumab | 7 | 1 |
| **161** | Itolizumab | 15.8 | 1 |
| **162** | Fremanezumab | 1.6 | 0 |
| **163** | Enfortumab | 1 | 0 |
| **164** | Emicizumab | 3.5 | 1 |
| **165** | Emapalumab | 3.5 | 1 |
| **166** | Efalizumab | 6.3 | 1 |
| **167** | Crizanlizumab | 0.9 | 0 |
| **168** | Cemiplimab | 1.3 | 0 |
| **169** | Brodalumab | 3 | 1 |
| **170** | Bezlotoxumab | 0 | 0 |
| **171** | Belimumab | 2.4 | 1 |
| **172** | Afutuzumab | 6.6 | 1 |
| **173** | Obiltoxaximab | 2.5 | 1 |
| **174** | Actoxumab | 0 | 0 |
| **175** | Tosatoxumab | 2.1 | 1 |
| **176** | Derlotuximab | 33.3 | 1 |
| **177** | Edrecolomab | 80 | 1 |

*: At a threshold of 2%, samples with an Immunogenicity value higher than 2% are defined as high immunogenicity and labeled as 1, while other samples are defined as low immunogenicity and labeled as 0.
