# Supplementary material for: AbImmPred: An immunogenicity prediction method for therapeutic antibodies using AntiBERTy-based sequence features
Source: PLoS One. 2024 Feb 23;19(2):e0296737. doi: 10.1371/journal.pone.0296737 (PMC10889861; doi:10.1371/journal.pone.0296737)
Supplement: S2 Table — (DOCX) [file pone.0296737.s003.docx]

**S2 Table**. **The names and immunogenicity values of 22 therapeutic antibody samples in the independent test dataset.**

| **No.** | **Therapeutic antibody** | **Immunogenicity value (%)** | **Immunogenicity label** |
| --- | --- | --- | --- |
| **1** | Elotuzumab | 18.5 | 1 |
| **2** | Gemtuzumab | 0 | 0 |
| **3** | Idarucizumab | 4 | 1 |
| **4** | Ixekizumab | 8.5 | 1 |
| **5** | Vedolizumab | 9.5 | 1 |
| **6** | Denosumab | 1 | 0 |
| **7** | Olaratumab | 3.5 | 1 |
| **8** | Secukinumab | 1 | 0 |
| **9** | Golimumab | 4 | 1 |
| **10** | Tocilizumab | 2 | 1 |
| **11** | Daratumumab | 0 | 0 |
| **12** | Reslizumab | 5 | 1 |
| **13** | Inotuzumab | 2 | 1 |
| **14** | Ocrelizumab | 1 | 0 |
| **15** | Sarilumab | 3.8 | 1 |
| **16** | Tildrakizumab | 6.5 | 1 |
| **17** | Evolocumab | 0.3 | 0 |
| **18** | Dupilumab | 4 | 1 |
| **19** | Burosumab | 5 | 1 |
| **20** | Benralizumab | 13 | 1 |
| **21** | Alirocumab | 3.9 | 1 |
| **22** | Mepolizumab | 13 | 1 |
